# Contextual consistency promotes visual-haptic simultaneity perception

**DOI:** 10.3389/fnhum.2025.1550231

**Published:** 2025-03-11

**Authors:** Hiroyuki Umemura, Sunao Iwaki

**Affiliations:** ^1^Human Augmentation Research Center, National Institute of Advanced Industrial Science and Technology, Kashiwa, Japan; ^2^Human Informatics and Interaction Research Institute, National Institute of Advanced Industrial Science and Technology, Tsukuba, Japan

**Keywords:** multimodal integration, causality, vision, haptics, simultaneity

## Abstract

In this study, we investigate the influence of causality validity in the information provided to each of two sensory modalities on the integration of multisensory information. For the purpose, stimuli that simulated a causal event, a ball striking an object, were created using a head-mounted display and a haptic device. The visual position and motion of the object were aligned to the haptic feedback received by the observer. The haptic device delivered a vibration around the moment of impact. Three vibration directions were used to assess the effect of the validity of the causal relationship between the two events. Participants were asked to determine whether the collision of the ball and the vibration were simultaneous. The findings revealed that the participants were more likely to perceive the events as simultaneous when the direction of the vibration matched the ball’s movement. These results suggest that valid causal consistency across different modalities enhances the binding of these signals as originating from a single source.

## 1 Introduction

Humans perceive an event occurring in the external world through multiple modalities, such as vision, audition, and haptics. There may be no explicit markers indicating that signals in separate modalities arise from the same source, yet we naturally perceive these signals as originating from a common origin. The ventriloquism effect, in which the perceived location of a sound source shifts toward the location of a corresponding visual stimulus, exemplifies how the brain combines information from different modalities (Bertelson and Radeau, 1981; Jackson, 1953; Wallace et al., 2004). The extensively studied spatial ventriloquism effect also appears in combinations of visual and somatosensory signals and auditory and tactile signals (Caclin et al., 2002; Pick et al., 1969).

A similar effect in intersensory binding occurs in the temporal domain, known as the temporal ventriloquism effect (Bertelson and Aschersleben, 2003; Morein-Zamir et al., 2003). In temporal ventriloquism, the temporal characteristics of a visual stimulus, such as its onset, interval, or duration, can be altered by slightly asynchronous auditory stimuli. A clear example of temporal ventriloquism occurs when an abrupt sound causes the apparent onset of a slightly asynchronous flash to be perceived synchronous. Similar to spatial ventriloquism, temporal ventriloquism can be induced between touch and vision or touch and audition (Bresciani and Ernst, 2007; Keetels and Vroomen, 2008). The brain tolerates slight delays between two signals to bind them as if they originated from a single source. These delays, often referred to as the temporal (binding) window, would be allowed for efficient integration. The temporal window size could be modulated by the implicit knowledge about the extent to which signals originate from the same source. Welch and Warren (1980) termed this knowledge the ‘unity assumption’: An observer will more likely infer that the sensory inputs have a common origin when he or she has a strong belief. The strength of the unity assumption depends on several factors, including the spatiotemporal closeness between two signals, the cognitive factors discussed below, and/or experimental settings such as given instruction (Warren et al., 1981).

Previous research has experimentally confirmed the cognitive factors which influence multimodal integration, in other words, which strengthen the unity assumption in the temporal domain. Vatakis and Spence (2007) reported the role of cognitive factors with audiovisual speech stimuli. Their studies revealed that gender-congruent combinations of voices and faces led to increased multisensory binding. However, their subsequent studies (Vatakis et al., 2008; Vatakis and Spence, 2008) did not find differences between matching or mismatching call types of monkeys, nor differences between stimuli composed of matched visual images of an action and sound (e.g., smashing an ice block or bouncing a ball) and mismatched stimuli (e.g., the visual signal of a hammer smashing a block of ice and the sound of a ball bouncing). These studies suggest a unique characteristic of intersensory pairing in audiovisual speech.

Furthermore, intention or causality has been shown to affect the perception of the temporal relationship between two events. Haggard (2005) and Haggard et al. (2002) demonstrated that the perceived timing of intentional actions (e.g., key press) and their sensory consequences were drawn together in consciousness, making voluntary movements appear to occur later and their consequences earlier than in reality. This ‘intentional binding’ suggests the brain links intentional actions with their outcomes to construct a coherent conscious experience. Similarly, studies have indicated that even non-intentional (passive) actions (Borhani et al., 2017; Buehner, 2015; Suzuki et al., 2019) or machine-generated actions and their outcomes can lead to temporal binding (Buehner, 2012), indicating the effect of causality on temporal attraction.

While temporal binding in the visual–auditory domain and between intention and its outcome has been extensively investigated, research on the cognitive effects on temporal visual-haptic signal integration remains scarce. Maselli et al. (2016) demonstrated that cognitive factors such as body ownership can alter visual-haptic binding. During the body ownership illusion, the temporal window for integrating touch on a physical body with visual touch on a virtual body widened. Furthermore, the extent of this temporal window positively correlated with the intensity of the illusory experience of owning the virtual body. Here, we believe that more study is needed on how such cognitive factors modify the temporal integration of vision and touch. The current study was designed to explore the impact of cognitive factors on the integration of visual-tactile information in the temporal domain. Specifically, the study investigated the congruency between visual context and haptic information on multimodal temporal binding. This experimental situation was similar to the investigation in the visual–auditory domain conducted by Vatakis and Spence (2008), where they did not find a cognitive effect on binding. However, whether this finding holds in the visual-haptic domain has not been investigated. Vatakis and Spence (2008) investigated the integration of visual and auditory signals, where the context binding a visual event with an auditory event involved an impact sound—such as a hammer striking ice or fingers strumming guitar strings. The incongruent stimuli in their study were created by swapping sounds between different types of events. Following their experimental paradigm, we designed a scenario in which a visual stimulus (a ball dropping from above and striking a surface) causes a haptic effect (a haptic device vibrates due to the ball’s impact). In this scenario, the direction of the vibration should align with the ball’s trajectory; it cannot be horizontal or in-depth. Consequently, we introduced incongruent stimuli with vibrations in different directions. If the validity of the context influences temporal binding, we would expect to observe more frequent binding of visual and haptic signals in trials where the vibration direction is vertically aligned, consistent with the falling ball. This effect was tested using a haptic device capable of delivering designed haptic stimuli synchronized with the refresh timing of the visual display.

## 2 Experiment 1

### 2.1 Method

#### 2.1.1 Participants and ethic statements

Eleven participants, aged between 20 to 37 years (6 females; mean age ± SD: 25.4 ± 6.8), who were unaware of the experiment’s purpose, participated in the present study. All the experimental procedures received approval from the Ethics Committee for Human and Animal Research of the National Institute of Advanced Industrial Science and Technology (AIST). All the participants provided written informed consent in accordance with the Declaration of Helsinki before engaging the experiment. The required sample size for each experiment was determined based on a power analysis for a 3 × 9 repeated measures ANOVA (*F*-tests), considering two within factors: ‘vibration direction’ with three levels and ‘SOA’ with nine levels. Regarding the effect size, the referenced previous studies did not report effect sizes or provide standard deviations for estimation. Therefore, we adopted Cohen’s medium effect size (*f* = 0.25, Cohen, 1992) as a reference. Other parameters were set as follows: *α* = 0.05, (1−*β*) = 0.80, a correlation among repeated measures of 0.5, and a non-sphericity correction of 0.7. Based on these parameters, G*Power (Faul et al., 2007) estimated a required sample size of 10, which aligns with the sample sizes reported in the referenced previous studies, such as those by Keetels and Vroomen (2008) and Spence et al. (2003). Considering the possibility of non-participation or unusable data, 11 participants were recruited.

#### 2.1.2 Apparatus

**Figure 1:**
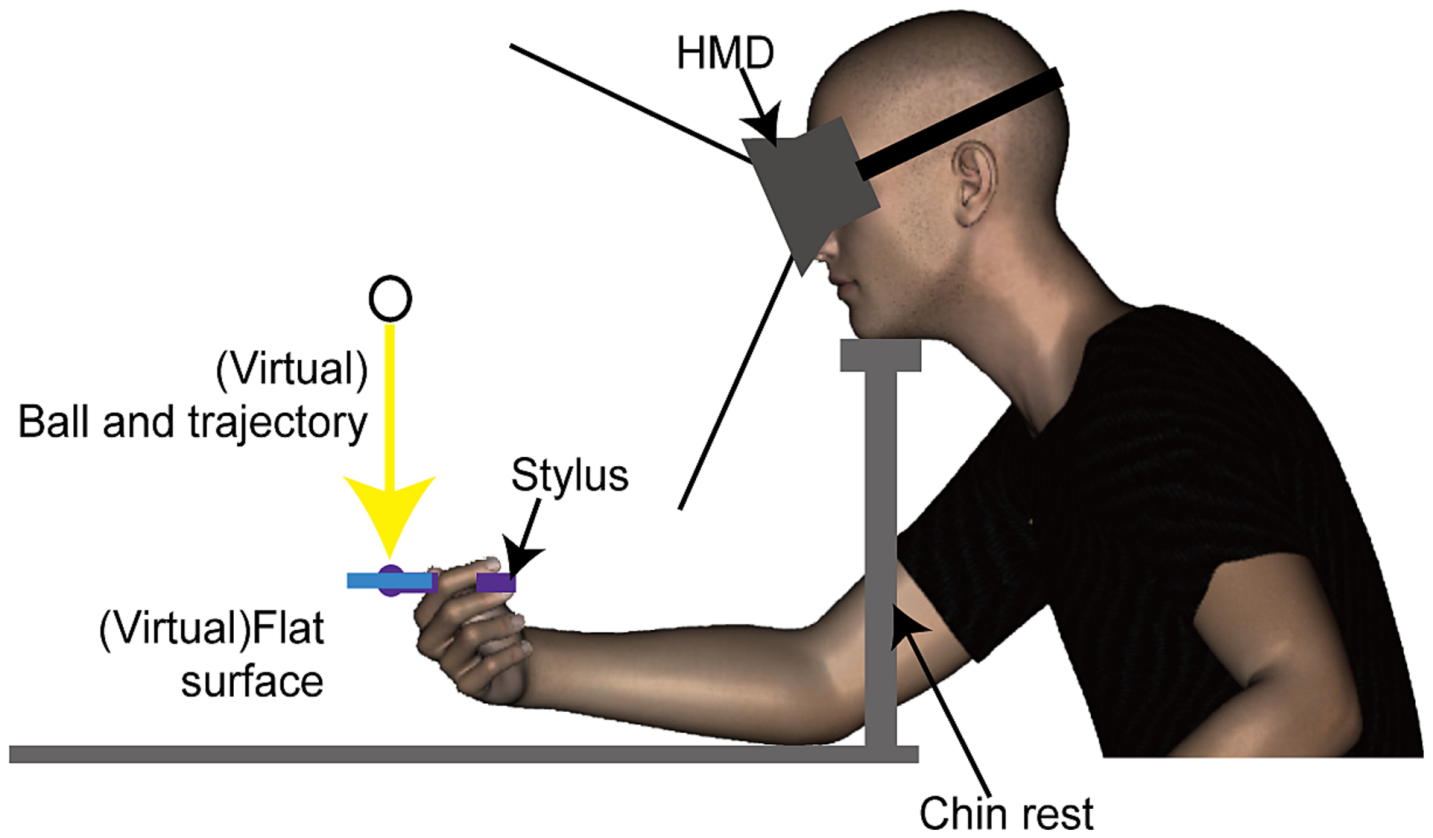
Side view of the experimental setup and virtual environment used in Experiment 1. Participants viewed a three-dimensional stimulus through a head-mounted display and received a haptic stimulus via a stylus connected to a haptic device (refer to Figure 2 for additional details).

A head mounted display (HMD, Oculus DK1, Oculus Inc.) was used to display stimuli. This HMD features a resolution of 1,280 × 800 pixels (640 × 800 per eye for binocular viewing) and operates at a refresh rate of 60 Hz. Although the HMD can detect and adjust to its tilting angles (pitch, yaw, roll), participants’ head movements were restricted using a chinrest. This procedure ensured a consistent distance between the HMD and the haptic device (Figure 1). Tactile stimuli were delivered using a haptic device (Phantom Desktop, Sensable Technologies). This device is designed to apply force in a programmed direction at a refresh rate of 1 kHz through a stylus held by the participant, and could synchronized with display redrawing timing. The position of the stylus was tracked by a computer for synchronizing the position and movement of an object in the visual scene with the participant’s hand movements.

#### 2.1.3 Stimuli

The stimuli used in this study were composed of visual and haptic elements. The visual component consisted of computer-generated animations showing a ball moving vertically toward a rectangular object. This object’s position was synchronized with the position of the haptic device’s location. The animations were rendered in three dimensions and presented to participants binocularly. The visual stimuli were scaled to match real-world dimensions. The ball, depicted as a shaded green sphere, measured 1 cm in diameter and traveled a 20 cm vertical path at a constant speed towards the center of a flat rectangular object. This object’s center was aligned with the haptic stylus’s point, set 30 cm from the chinrest (Figure 1). Should the stylus deviate slightly from its fixed position, the ball’s position and trajectory would adjust accordingly, ensuring it always moved vertically towards the flat surface’s center. The ball’s motion, starting from rest, took 500 milliseconds to reach the object and stopped upon contact.

**Figure 2:**
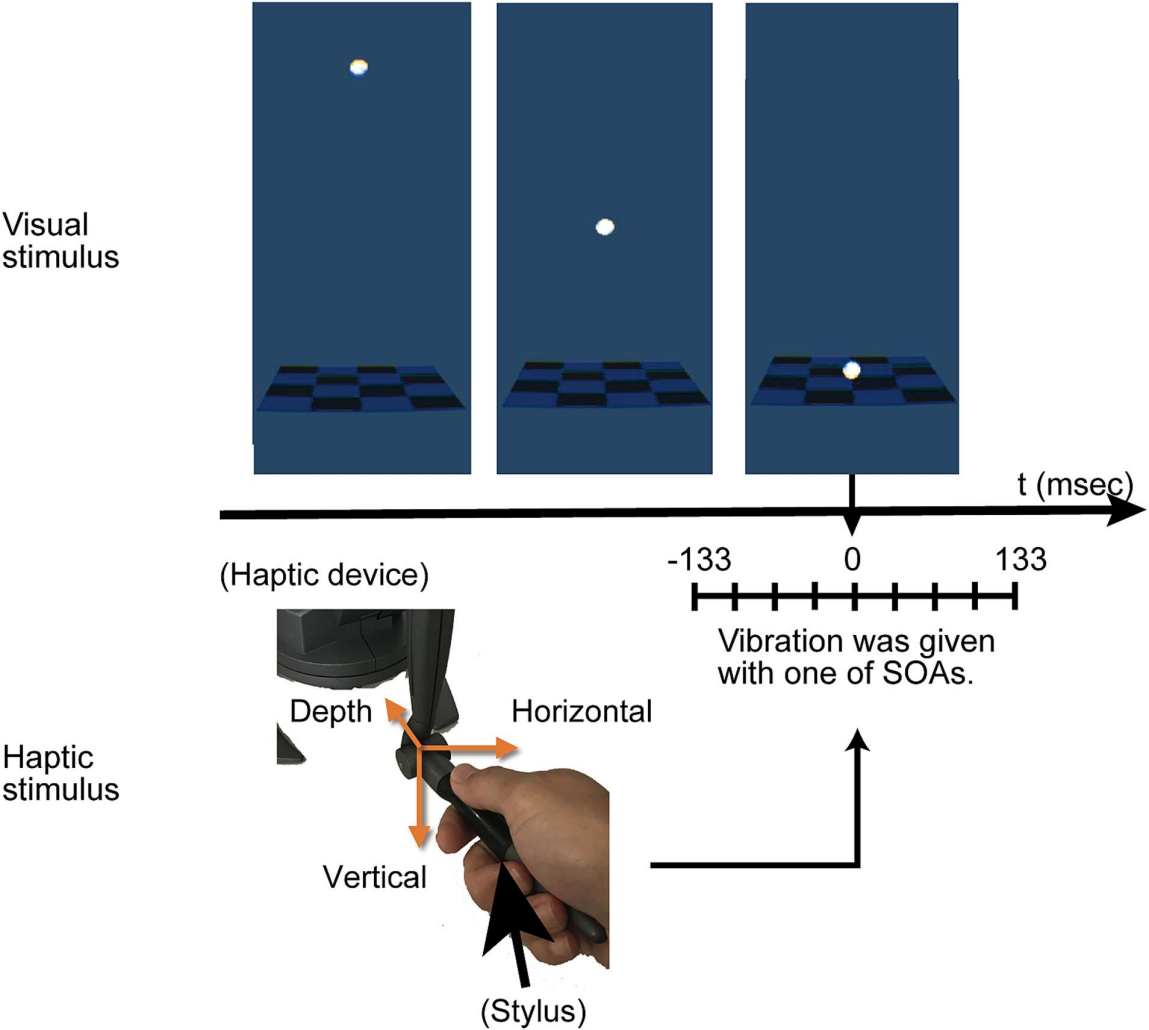
Overview of the experimental procedure. The visual stimulus involved a white ball moving downward and stopping on a flat surface, aligned with the position of the stylus. A haptic vibration in one of three directions, vertical, horizontal or in depth, was delivered around the moment the ball reached the plane depicted at the tip of the stylus. There were slight asynchrony between the visual contact of the ball and the vibration, and varied from −133 (negative value indicates haptics first) to 133 ms. Participants were asked to answer whether the visual and haptic events occurred simultaneously.

The haptic stimuli comprised vibrations transmitted through the stylus held by participants. These vibrations had a 5 mm amplitude and occurred at a 1/100-s cycle. They were directed in one of three orientations: vertical, horizontal, or in-depth (Figure 2). Although the vibrations were subtle, identifying their directions was relatively easy if observers were taught the existence of three directions in advance. We recruited three participants without involvement in the current experiment to test direction identification. After experiencing the vibrations with feedback, each participant was presented with the direction of the vibration 10 times in a random order. All participants were able to identify the directions accurately. The Stimulus Onset Asynchrony (SOA) between the stop of the ball’s movement and the onset of vibration varied at −133, −100, −67, −33, 0, 33, 67, 100, or 133 ms. The negative SOA values indicated that the vibration initiated before the ball contacted the rectangular object. For instance, at an SOA of −133 ms, the vibration was triggered when the ball was 53 mm above the surface.

#### 2.1.4 Procedure

The experiment was conducted in a dimly lit room. Participants wore a head-mounted display (HMD) and headphones, nullifying the haptic device’s operational noise. Seated at a table, all participants (right-handed) held the stylus of the haptic device with their right hand. They were informed that the displayed rectangular object aligned with the stylus’s position and would follow its movements. The participants were allotted time to familiarize themselves with this setup by moving the stylus independently.

Participants were required to align the hand (holding the stylus) at the center of the display and maintain this position throughout the stimulus presentation. While the vibrations were delivered in alignment with external coordinates regardless of the stylus’s orientation, it was essential for its axis to remain horizontal to the ground and perpendicular to the body. Participants were allowed to rest their elbows on the table but were instructed to keep their palms elevated (Figure 1).

Each trial commenced with the participant pressing a button on a haptic device with thumb finger (The button is placed under the thumb; Figure 2). Following the display of the visual collision and vibration, participants used a keyboard to indicate whether they perceived the events (the visual collision and the vibration) as simultaneous (‘5’ key) or not (‘8’ key). This keyboard was accessible to their left hand. The participants were required to gaze at the center of the rectangular surface, i.e., contact position. Before the official experimental sessions began, participants engaged in practice trials. In these trials, each participant experienced every combination at least once, resulting in 27 trials in total. Participants had the option to extend the practice period until they felt satisfied.

In Experiment 1, participants underwent three sessions. Each session involved displaying one of three vibration directions with nine different SOAs, presented in random order and repeated thrice;. Therefore, each combination of vibration and Stimulus Onset Asynchrony (SOA) was presented nine times per participant. Each session lasted 15 to 25 min. Breaks of 5 or 10 min were given between them. An experimenter monitored the visual stimuli presented in the HMD through an external display in the same room, one partition away. Trials where participants accidentally pressed the incorrect button were reported to the experimenter in the same room, and excluded from subsequent analysis. One participant who reported mistakes more than five times in the first session underwent an additional session, and the records from the first session were discarded. Upon finishing all experimental procedures, participants were questioned regarding their awareness of the variation in vibration direction across trials and whether this knowledge influenced their judgment of simultaneity.

### 2.2 Results of Experiment 1

**Figure 3:**
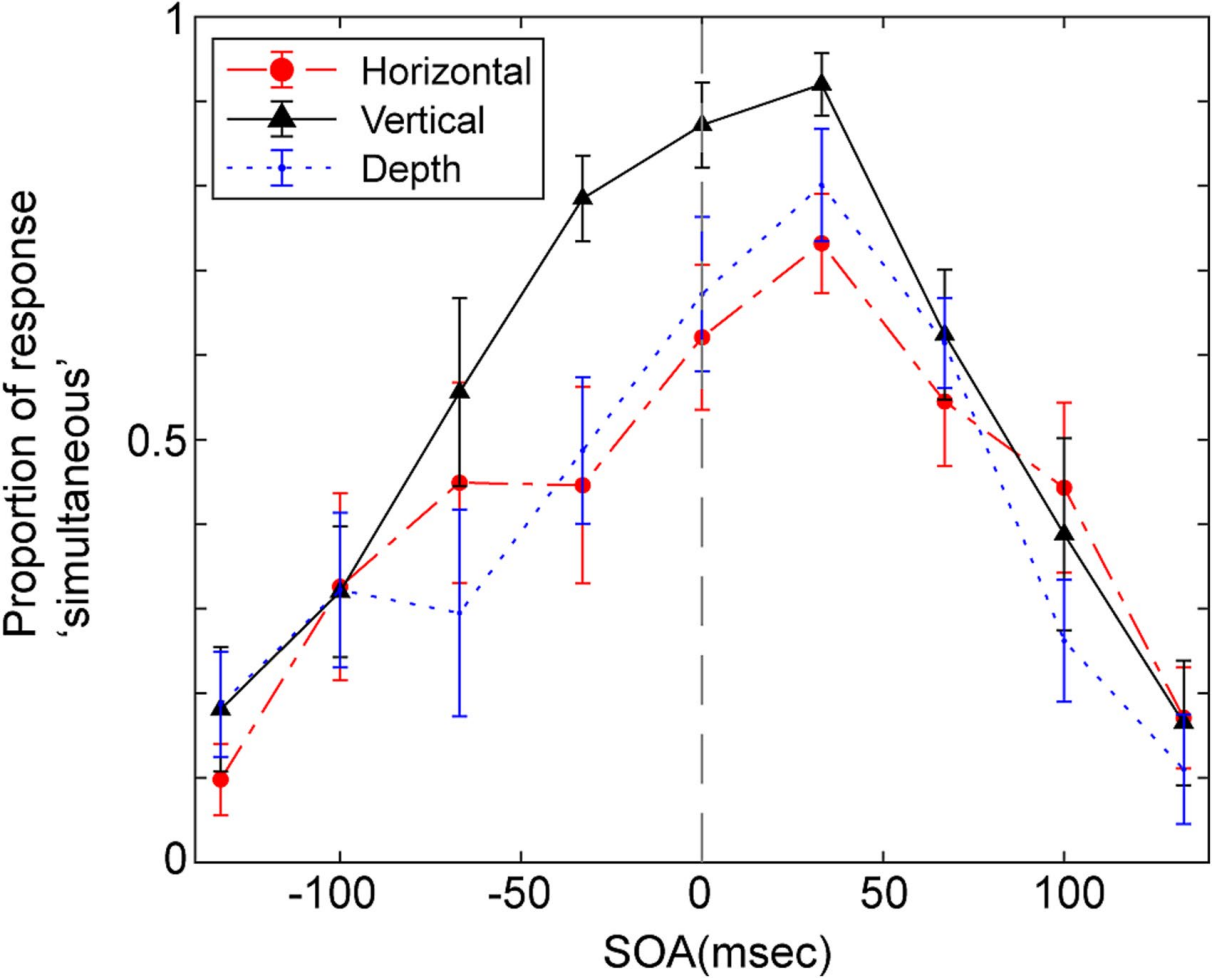
Mean proportion of response “simultaneous” as a function of SOA for the simultaneity judgments in Experiment 1. Positive SOA indicates visual stimulus comes first and the haptic stimulus. Error bars represent the standard error of the mean.

Figure 3 displays the average probability of participants responding ‘simultaneous’ as a function of the SOA for the three vibration directions. A repeated two-way ANOVA was conducted, focusing on within-participant factors of SOA and the direction of vibration to assess their impact on judgments of simultaneity. The Huynh-Feldt epsilon correction was applied to evaluate F ratios for repeated measures with more than one degree of freedom. The analysis yielded significant main effects for SOA [*F* (3.67, 36.68) = 27.38, *p* < 0.001, *ε* = 1.0, partial η^2^ = 0.733], and the direction of vibration [*F* (2.00, 20.00) = 17.47, *p* < 0.001, ε = 0.46, partial η^2^ = 0.636], as well as a significant interaction between these two factors [*F* (11.62, 116.19) = 2.27, *p* = 0.014, ε = 0.73, partial η^2^ = 0.185].

Multiple pairwise comparisons, applying the Holm-Sidak correction, were performed across combinations of vibration direction and SOA. These comparisons indicated significant differences in several SOAs, particularly between the vertical direction and the other two directions (depth and horizontal). Notably, significant differences (*p* < 0.05) were found in vertical and depth directions comparisons at SOAs of −67, −33, and 0 ms. Significant differences emerged in comparisons between vertical and horizontal directions at SOAs of −33, 0, and 33 ms. In all these cases, the probability of judging events as simultaneous was higher when the vibration direction was vertical, though the significant differences were seen in different SOAs.

**Figure 4:**
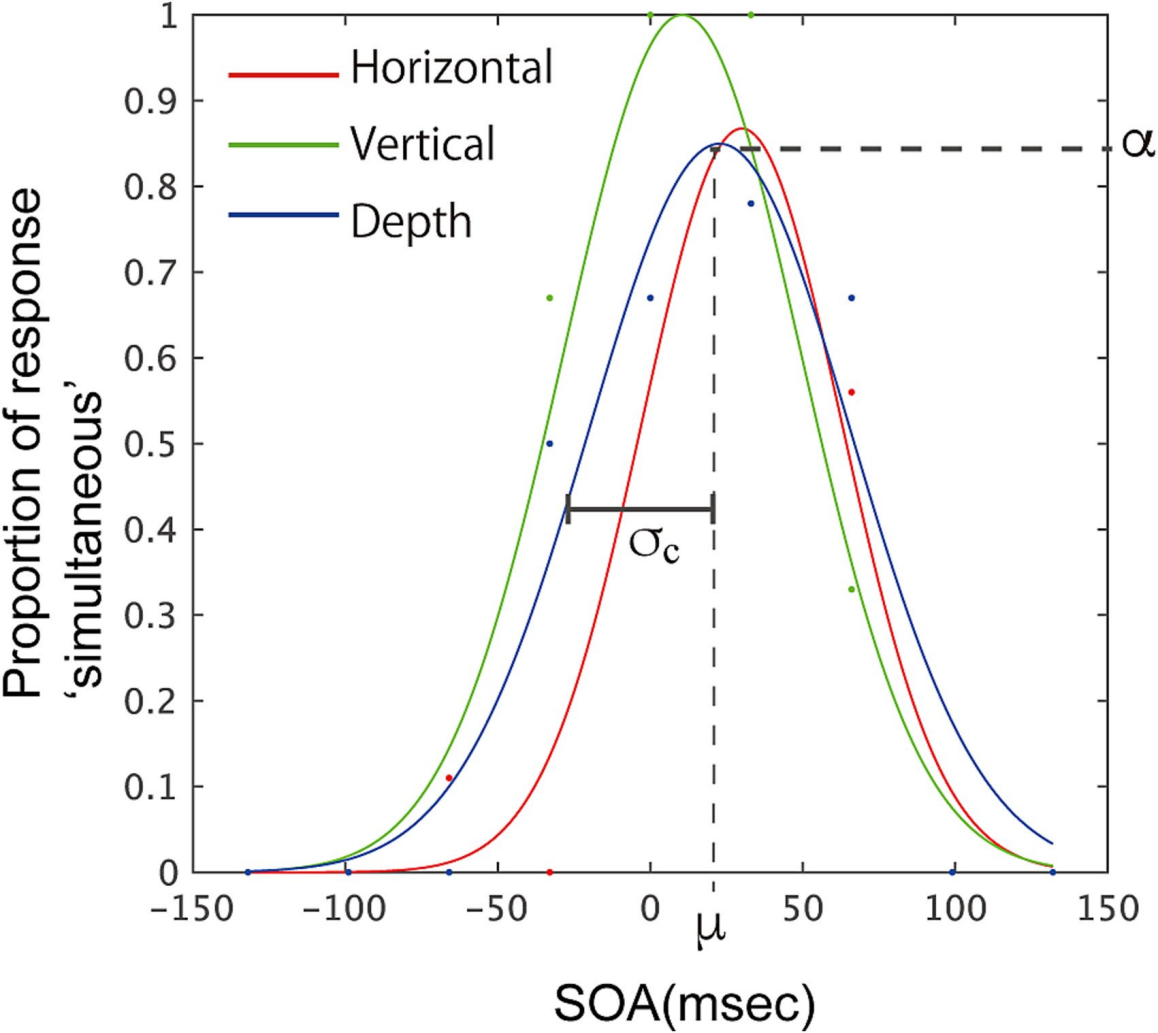
Example of fitted Gaussian curves, f = α*exp.(−((x−*μ*)/*σ*)^2^), for one participant, illustrating parameters for the fitting. Data points from the experiment are marked with dots. The illustrated parameters correspond to the fitting for the depth-vibration condition (blue curve). Parameters α, σ, and μ were estimated for the Gaussian fitting. σ_c_ is the product of σ and α, and corresponds to the width of the temporal window.

We conducted further analysis by fitting a Gaussian curve, f = α*exp.(−((x−*μ*)/*σ*)^2^), to the proportion of simultaneous responses obtained from each participant, estimating three optimal parameters: α, μ, and σ. α represents the height of the Gaussian (≦1.0) and indicates the highest probability of simultaneous responses. The mean of the Gaussian, μ, corresponds to the accuracy of the participants’ judgments when integrating information from two modalities. The width of the Gaussian, σ, correspond to the sensitivity of the individual responses. At the same time, σ correspond to the tolerance for binding two signals, but the range along SOA covered by an estimated Gaussian with certain σ becomes smaller as α for the Gaussian becomes smaller. This does not match the intuitive window size for the integration, so we compensated the value by calculating the product of σ and α. This value, σ_c_ is also obtained, and used in the following analysis. With these parameters, we could visualize individual responses. The fitting and calculation were performed using functions in MATLAB (MathWorks, Inc.). Figure 4 illustrates the fitted Gaussian curves for one participant and displays the estimated parameters.

**Figure 5:**
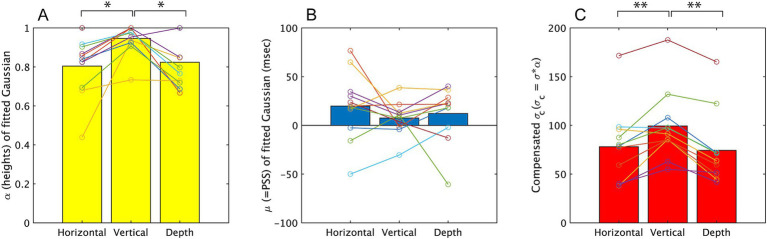
The parameters obtained from fitting a Gaussian, f = α*exp.(−((x−μ)/σ)^2^), to the results of Experiment 1. The mean value among all participants is represented by a bar graph, and the estimated values for each subject are indicated by circles. Results on α, μ, σ_c_ are displayed on **A–C**, respectively. *p < 0.05 and **p < 0.01.

Figure 5 summarizes the parameters obtained from the fitting. This figure includes the estimated parameters for each observer and their averages among observers. We also calculated the *R*-square (*R*^2^) which indicates the quality of the fit of the Gaussian curve (see Supplementary material). The mean *R*^2^ of both participants and conditions was 0.80 and this suggests a moderate fit of the Gaussian curve, while a relatively large standard deviation points to significant individual differences among participants (see Supplementary material). Specifically, averaged *R*^2^ values among three directions for each participant ranged from 0.58 to 0.95, suggesting that the task might be difficult for some participants.

The one-way ANOVA on the three directions revealed a significant effect of direction on *α* [*F* (2, 20) = 6.29, *p* = 0.005, partial η^2^ = 0.689]. Multiple pairwise comparisons using the Holm-Sidak correction indicated significant differences between the vertical vibration condition and the other two directions (*p* = 0.016 for horizontal direction and *p* = 0.022 for depth direction). These differences suggest that participants more frequently responded ‘simultaneously’ in the vertical-vibration condition, as observed in the analysis for the response probability done in above. The one-way ANOVA for *σ* (see Supplementary material for σ values) did not show a significant effect of direction [*F* (2, 20) = 2.483, *p* = 0.109, partial η^2^ = 0.199], while σ_c_ showed a significant effect of direction [*F* (2, 20) = 15.55, *p* < 0.001, partial η^2^ = 0.609]. Multiple pairwise comparisons using the Holm-Sidak correction for the latter indicated significant differences between the vertical direction and the depth direction (*p* < 0.001) and the difference between vertical and horizontal direction (*p* = 0.007). The larger σ_c_ in the vertical vibration direction suggests that the temporal window was larger than other two directions in this condition. While σ were not significantly different among the three directions, indicating that the congruency of the visual and haptic directions uniformly enhanced the simultaneous judgment along SOAs.

The ANOVA for *μ* did not show significant difference [*F* (2, 20) = 0.884, *p* = 0.429, partial η^2^ = 0.081] among three directions. Here, we also concerned about whether μ were significantly different from 0 ms, therefore we conducted *t*-test to examine this. The results of analysis showed no significant differences from 0 in all three directions [*t* (10) = 1.701, *p* = 0.123, *t* (10) = 1.313, *p* = 0.218, *t* (10) = 1.215, *p* = 0.252, for horizontal, vertical, depth directions respectively].

### 2.3 Discussion of Experiment 1

The findings of Experiment 1 demonstrate that the direction of vibration significantly influences simultaneous judgments. Notably, when the vibration direction matched the direction of the ball’s movement in the visual modality, both the likelihood and the range of simultaneous judgments increased. This aligns with the hypothesis that consistent causal contexts between visual and haptic modalities facilitate the integration of these signals, perceived as emanating from a single source, such as the vibration caused by the ball’s collision. Of course, it is necessary to consider why the results observed here were not seen in the audio and visual integration. This will be addressed in the General Discussion.

Participants often judged the events as asynchronous even at an SOA of 0 ms when the vibration direction was incongruent (i.e., horizontal or depth direction) with the visual contact; they did not attain 0.7 at 0 ms, and the maximum probability in these two directions was 0.8 in 33 ms SOA. This would indicate that spatial discrepancies in sensory processing can significantly deteriorate simultaneity judgments.

The peak of simultaneous judgments seems slightly shifted toward positive SOA in Figure 3 which indicates that participants often perceived simultaneous when the tactile sensation occurred slightly after the visual collision. Such a shift is reported in previous studies in vision-haptics or vision-audition integration (Harrar and Harris, 2005; Arrighi et al., 2006). The ANOVA for estimated *μ*, however, did not show the significant difference.

In summary, the results of Experiment 1 support the notion that a causal relationship between visual and haptic modalities enhances signal binding. However, the possibility remains that the observed results in Experiment 1 could be specific to the vertical direction of the stimuli. Suppose the causal relationship between the visual collision and the direction of vibration promoted binding these two events, similar results should be obtained even when the direction of the ball’s trajectory is changed.

## 3 Experiment 2

### 3.1 Apparatus and stimuli

**Figure 6:**
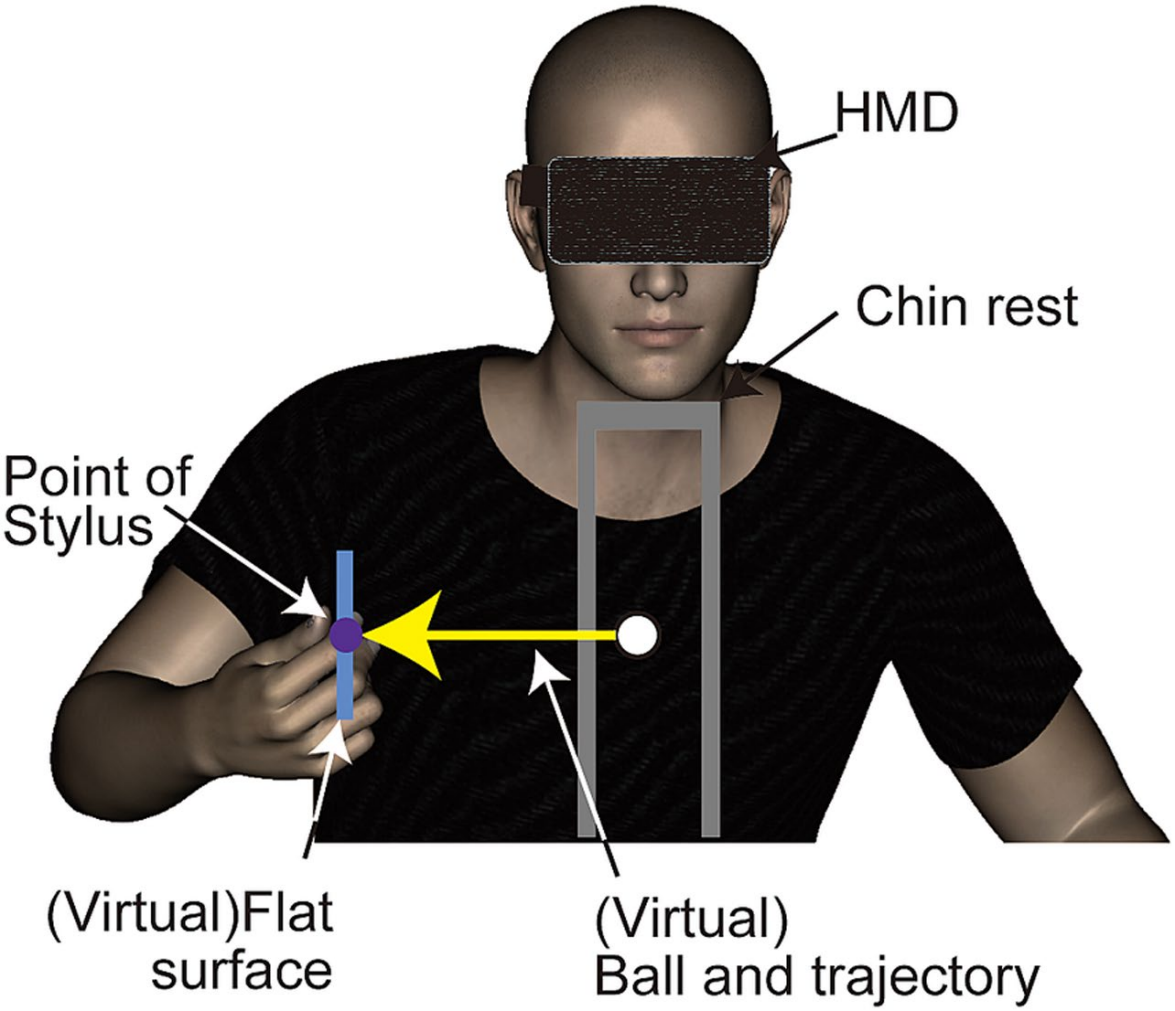
Front view of the experimental setup and virtual environment used in Experiment 2. In this setup, the ball moved horizontally, and the subjects held the stylus so that the tip of the stylus would align with the ball’s trajectory. As in Experiment 1, around the time the ball reached the plane depicted at the tip of the stylus, the stylus vibrated in one of three possible directions.

Experiment 2, depicted in Figure 6, introduced a variation in the stimuli setup of Experiment 1; the ball moved horizontally. Participants were instructed to position the stylus 15 cm to the right of the mid-line and 15 cm above the table. Similar to Experiment 1, if the stylus deviated slightly from its designated position, the ball’s position and trajectory would adjust accordingly, ensuring it always moved horizontally towards the center of the flat surface. The required orientation of the stylus remained the same: horizontal to the ground and perpendicular to the body. Participants lifted their elbows from the table because it was more comfortable in this setup. All other aspects, including the apparatus and procedure, were identical to those in Experiment 1. We collected 11 participants who were not participated in Experiment 1. Although the result of one participant showed that the participant did not understand the instruction, we used data from 10 participants (7 females; mean age ± SD: 27.3 ± 6.0) in the analysis because the sample size attained the size calculated in the power analysis (see Section 2.1.1).

### 3.2 Results of Experiment 2

**Figure 7:**
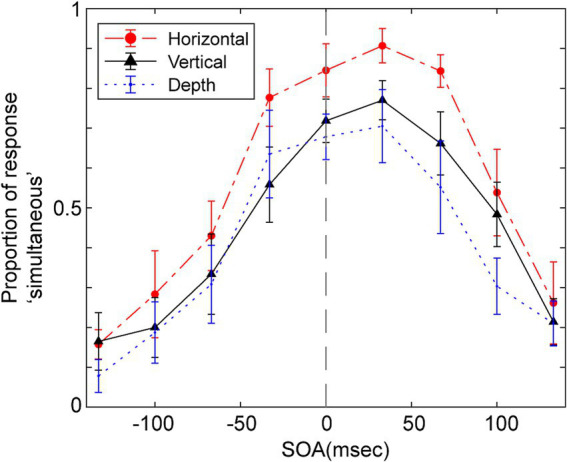
Mean proportion of response ‘simultaneous’ as a function of SOA for the simultaneity judgments in Experiment 2. Error bars represent the SEM.

Figure 7 presents the average probability of simultaneous responses among participants in relation to the SOAs for the three vibration directions. A repeated two-way ANOVA was conducted with SOA and vibration direction as within-participant factors. As in the Experiment 1, the Huynh-Feldt epsilon correction was applied to evaluate F ratios. The analysis revealed a significant main effect for SOA [*F* (5.5, 49.78) = 25.9, *p* < 0.001, *ε* = 1.0, partial η^2^ = 0.742], and the direction of vibration [*F* (2.00, 18.00) = 10.9, *p* < 0.001, ε = 0.69, partial η^2^ = 0.548], but no significant interaction was found [*F* (13.40, 120.61) = 0.767, n.s., ε = 0.84, partial η^2^ = 0.079]. Using the Holm-Sidak correction, multiple pairwise comparisons were performed across combinations of vibration direction and SOAs. These comparisons showed that significant differences (*p* < 0.05) were present at an SOA of 0 ms between the horizontal and vertical directions and between the horizontal and depth directions.

**Figure 8:**
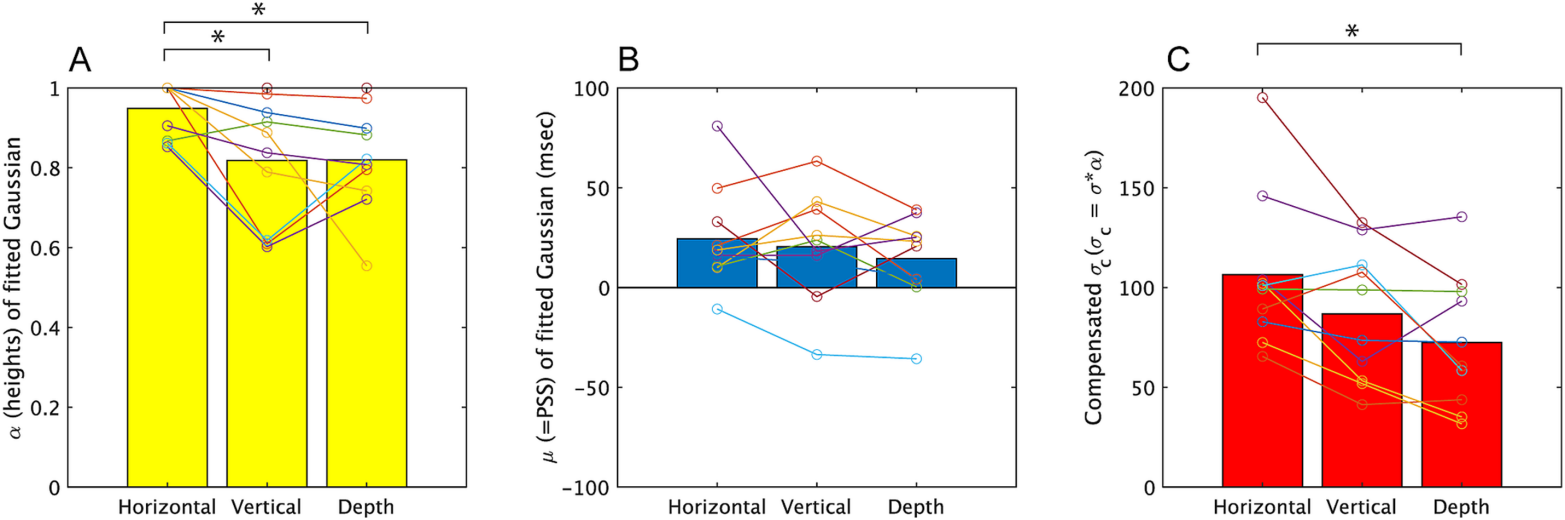
The parameters obtained from fitting a Gaussian, f = α*exp.(−((x−μ)/σ)^2^), to the results of Experiment 2. The mean value among all participants is represented by a bar graph, and the estimated values for each subject are indicated by circles. σ_c_ is the product of σ and α, and corresponds to the width of the temporal window. Results on α, μ, σ_c_ are displayed on **A–C**, respectively. *p < 0.05 and **p < 0.01.

We conducted further analysis in Experiment 2, by fitting Gaussian curves. Figure 8 summarizes the parameters obtained from the fitting. The mean *R*^2^ values of both participants and conditions (see Supplementary material for detail) was 0.76 and this was slightly lower compared to those in Experiment 1. This would indicate the task was slightly difficult. The one-way ANOVA on the three directions revealed a significant effect of direction on *α* [*F* (2, 20) = 6.29, *p* = 0.005, partial η^2^ = 0.689]. Multiple pairwise comparisons using the Holm-Sidak correction indicated significant differences between the horizontal vibration condition and the vertical directions (*p* = 0.016), and depth direction (*p* = 0.022). As same as Experiment 1, the ANOVA for *σ* (see Supplementary material for these values) did not show significant difference, but the ANOVA for σ_c_ also showed a significant effect of direction [*F* (2, 20) = 9.11, *p* = 0.002, partial η^2^ = 0.477]. Multiple pairwise comparisons using the Holm-Sidak correction indicated significant differences between the horizontal direction and the depth direction (*p* < 0.009) but the difference between vertical and horizontal direction did not attain the significance level (*p* = 0.072). While, σ and *μ* showed no significant difference [*F* (2, 20) = 2.557, *p* = 0.105, partial η^2^ = 0.221, *F* (2, 20) = 0.895, *p* = 0.426, partial η^2^ = 0.090].

We conducted *t*-test to examine whether *μ* were significantly different from 0 ms also for the results of Experiment 2. The results of analysis showed significant differences from 0 in two of three directions [*t* (9) = 3.068, *p* = 0.013, *t* (9) = 2.429, *p* = 0.038, *t* (9) = 2.072, *p* = 0.068, for horizontal, vertical, depth directions respectively].

### 3.3 Discussion of Experiment 2

The findings of Experiment 2 demonstrated frequency of simultaneous judgments was also increased even when the direction of vibration was horizontal. Additionally, the temporal window was wider in the horizontal direction. In Experiment 2, the difference of estimated σ_c_ between vertical and horizontal vibration directions did not attain significant level. This may be caused from unstable hand pose in the setting of Experiment 2.

The results of Experiment 2 indicate that the facilitation of simultaneity perception when the direction given to the two modalities is congruent is not limited to the vertical direction. Thus, while Experiment 2 yielded results largely consistent with those of Experiment 1, a noteworthy distinction was observed: the estimated μ value, representing the timing of the tactile stimulus most likely perceived as simultaneous, was significantly delayed relative to the timing of visual contact. We will discuss the results in general discussion.

## 4 General discussion

This study demonstrates that congruency in the direction of visual motion and hand-delivered vibration increases the frequency of ‘simultaneous’ responses concerning the timing of visual collision and haptic vibration. To understand the results of these experiments, it is necessary to focus on the aspect that causality can influence time perception, as well as on the importance of consistency of the causal events given to different modalities. Concerning the former, previous studies have indicated that such a causal relationship alters the temporal relationship between events within the visual domain (Bechlivanidis and Lagnado, 2016; Umemura, 2017), and in visual–auditory domains (Buehner and Humphreys, 2009; Kohlrausch et al., 2013; Fornaciai and Di Luca, 2020). For example, Fornaciai and Di Luca (2020) showed that the audio-visual stream-bounce stimuli induce perceptual delay in the audio component. This indicates they are interpreted to be consistent with a causal relation even if the sound was actually presented first. Our study shows that modifying this temporal relationship is contingent on the congruency of cause and effect even in the visual-haptic domain.

While concerning the consistency of the causal events, our results diverge from previous research on auditory and visual information integration (Vatakis and Spence, 2008), who investigated similar experimental settings and causal situations. The difference may arise from the less specificity of matched stimuli in the previous study, which perhaps did not create a substantial difference from mismatched stimuli to trigger temporal attraction. While the matched stimulus in our study, an object falling from above vibrating in a congruent direction, created a substantial difference from the mismatched stimulus. We speculate that this discrepancy could be due to the fact that both auditory and visual signals have external sources, whereas our experiment directly involved the body through tactile sensation. However, this interpretation contrasts with findings from another study that observed a unity effect with stimuli that combined a visual stream of mouth movements and gender-matched or mismatched voices (Vatakis and Spence, 2008). Following study by Vatakis et al. (2008) suggested that the facilitation of multisensory integration is specific to human speech. Vroomen and Stekelenburg (2011) speculated that a fine temporal correlation between sound and vision in speech contributes to this effect. Accepting these views implies that factors promoting temporal attraction are multifaceted, including stimulus congruency, presentation methods, and complexity.

The effect of orientation congruency was observed similarly in both vertical and horizontal directions. While horizontal movements may occur less frequently in daily visual experience compared to those in the direction of gravity, we believe that the brain has acquired enough samples to form prior knowledge. Furthermore, in the present experiment, tactile contact occurred simultaneously with visual observation. Considering real-life situations such as catching a ball, the frequency difference between directions may be further reduced. However, it remains possible that differences in ball-direction exert some influence on the observed effects. A comparison of the results from Experiments 1 and 2 reveals that while no peak shift was observed in Experiment 1, Experiment 2 exhibited a shift in the direction where haptic feedback followed the visual collision. For the lack of shift in Experiment 1, a possible explanation could be the brain’s expectation of the collision timing based on the falling ball’s direction. For example, in a vertical descent in Experiment 1, the brain might anticipate acceleration due to gravity affecting the perceived timing of collision (McIntyre et al., 2001; Senot et al., 2005; Zago et al., 2008), though the ball speed was constant in the present experimental setting. If the decisions were made with the expectation, the actual timing of visual collision is later than the expected timing and this canceled the shift caused by causality. Yet further investigation is required to substantiate this view. The number of participants and the number of SOA conditions might be too small to thoroughly investigate these topics here, although they were sufficient to demonstrate the existence of contextual effects in the visual-haptic domain. In future studies, expanding the number of participants and conditions would allow for a more precise estimation of the response curves’ shapes and facilitate a deeper discussion of their characteristics including the difference among different directions.

Previous studies have emphasized the significance of predictive processes in temporal binding, particularly in relation to a person’s actions or intentions (Hughes et al., 2013; Maselli et al., 2016; Waszak et al., 2012). In our study, alongside the predictive processes, the postdictive processes, where a later stimulus influences an earlier one, seems to play a crucial role, especially considering the unpredictability of the vibration direction. Such phenomena have been incorporated into models explaining temporal aspects of visual perception and multimodal integration, suggesting that conscious perception is shaped by unconscious processing that utilizes prior knowledge to reconstruct temporal relationships (Hogendoorn, 2022; Shimojo, 2014). It has been proposed a model to unify the computational principles of predictive and postdictive processes in the Bayesian framework of uncertainty reduction (Jagini, 2021). Bayesian framework would fit well to our results because causality can be represented as a prior knowledge and also fit well to the notion of the ‘unity assumption’ (Chen and Spence 2017).

One of limitation of the current study is that there was no condition in which the visual stimulus lacked directional information. As a result, we were unable to examine whether the facilitation effect arises because the brain perceives causality as established, or whether the lack of causality acts as an interfering factor. Regarding this issue, a previous study by Ho et al. (2009) reported that spatially incongruent sensory information from other modalities tends to be more disruptive to task performance compared to when such information is absent, while spatially congruent information produces a strong facilitation effect. Although their study focused on attentional allocation, it will be necessary in future research to experimentally investigate whether similar tendencies are observed in the present task.

Another concern is that two direction conditions were separately conducted. The experiment was conducted in accordance with the reported procedure, that is Experiment 2 was planned after Experiment 1. Furthermore, mixing two-direction conditions would have necessitated participants to adjust their hand position on a trial-by-trial basis, potentially increasing their physical load. To mitigate this issue, two direction conditions were separately conducted. Consequently, stimuli were presented in the same direction consecutively during the experiment, and we cannot entirely rule out the possibility that this repeated presentation in the same direction facilitated integration in a specific direction. As reported by Lange et al. (2018), recalibration in multisensory integration can occur on a trial-by-trial basis, suggesting that some degree of modulation may take place over a very short period. While it is unlikely that the main finding of this experiment would disappear if stimulus directions were mixed, it is important to consider such factors when evaluating the magnitude of the observed effects.

Finally, it is important to acknowledge that the method used to determine the sample size in this study was not based on empirical evidence for power estimation. Additionally, the reference experiment used as a guideline was relatively outdated, which should be taken into consideration. As mentioned earlier, redesigning the number of participants and conditions while considering further analyses and fitting would likely yield more detailed insights.

In this study, we demonstrated the influence of causality validity in the information provided to each of two sensory modalities on the integration of multisensory information. This finding underscores the need for further research to understand the various factors affecting multisensory integration and how the brain utilizes information to construct our perception of the external world. Future studies should also explore how prior information, such as causal relationships, is formed and used in the integration process.

## Data Availability

The original contributions presented in the study are included in the article/Supplementary material, further inquiries can be directed to the corresponding author.

## References

[ref1] Arrighi R. Alais D. Burr D. (2006). Perceptual synchrony of audiovisual streams for natural and artificial motion sequences. J. Vis. 6, 6–268. doi: 10.1167/6.3.6, PMID: 16643094

[ref2] Bechlivanidis C. Lagnado D. A. (2016). Time reordered: causal perception guides the interpretation of temporal order. Cognition 146, 58–66. doi: 10.1016/j.cognition.2015.09.001, PMID: 26402648

[ref3] Bertelson P. Aschersleben G. (2003). Temporal ventriloquism: crossmodal interaction on the time dimension. 1. Evidence from auditory-visual temporal order judgment. Int. J. Psychophysiol. 50, 147–155. doi: 10.1016/s0167-8760(03)00130-2, PMID: 14511842

[ref4] Bertelson P. Radeau M. (1981). Cross-modal bias and perceptual fusion with auditory-visual spatial discordance. Percept. Psychophys. 29, 578–584. doi: 10.3758/bf03207374, PMID: 7279586

[ref5] Borhani K. Beck B. Haggard P. (2017). Choosing, doing, and controlling: implicit sense of agency over somatosensory events. Psychol. Sci. 28, 882–893. doi: 10.1177/0956797617697693, PMID: 28488908

[ref6] Bresciani J. P. Ernst M. O. (2007). Signal reliability modulates auditory-tactile integration for event counting. Neuroreport 18, 1157–1161. doi: 10.1097/WNR.0b013e3281ace0ca, PMID: 17589318

[ref7] Buehner M. J. (2012). Understanding the past, predicting the future: causation, not intentional action, is the root of temporal binding. Psychol. Sci. 23, 1490–1497. doi: 10.1177/0956797612444612, PMID: 23104679

[ref8] Buehner M. J. (2015). Awareness of voluntary and involuntary causal actions and their outcomes. Psychol. Conscious. Theory Res. Pract. 2, 237–252. doi: 10.1037/cns0000068, PMID: 39780972

[ref9] Buehner M. J. Humphreys G. R. (2009). Causal binding of actions to their effects. Psychol. Sci. 20, 1221–1228. doi: 10.1111/j.1467-9280.2009.02435.x, PMID: 19732384

[ref10] Caclin A. Soto-Faraco S. Kingstone A. Spence C. (2002). Tactile “capture” of audition. Percept. Psychophys. 64, 616–630. doi: 10.3758/bf03194730, PMID: 12132762

[ref11] Cohen J. (1992). A power primer. Psychol. Bull. 112, 155–159. doi: 10.1037/0033-2909.112.1.155, PMID: 19565683

[ref9002] Chen Y-C. Spence C. (2017). Assessing the Role of the ‘Unity Assumption’ on Multisensory Integration: A Review. Front. Psychol. 8, 445. doi: 10.3389/fpsyg.2017.00445, PMID: 28408890 PMC5374162

[ref12] Faul F. Erdfelder E. Lang A.-G. Buchner A. (2007). G*power 3: a flexible statistical power analysis program for the social, behavioral, and biomedical sciences. Behav. Res. Methods 39, 175–191. doi: 10.3758/BF03193146, PMID: 17695343

[ref13] Fornaciai M. Di Luca M. (2020). Causality shifts the perceived temporal order of audiovisual events. J. Exp. Psychol. Hum. Percept. Perform. 46, 890–900. doi: 10.1037/xhp0000754, PMID: 32352821

[ref14] Haggard P. (2005). Conscious intention and motor cognition. Trends Cogn. Sci. 9, 290–295. doi: 10.1016/j.tics.2005.04.012, PMID: 15925808

[ref15] Haggard P. Clark S. Kalogeras J. (2002). Voluntary action and conscious awareness. Nat. Neurosci. 5, 382–385. doi: 10.1038/nn827, PMID: 11896397

[ref16] Harrar V. Harris L. R. (2005). Simultaneity constancy: detecting events with touch and vision. Exp. Brain Res. 166, 465–473. doi: 10.1007/s00221-005-2386-7, PMID: 16028031

[ref17] Ho C. Santangelo V. Spence C. (2009). Multisensory warning signals: when spatial correspondence matters. Exp. Brain Res. 195, 261–272. doi: 10.1007/s00221-009-1778-5, PMID: 19381621

[ref18] Hogendoorn H. (2022). Perception in real-time: predicting the present, reconstructing the past. Trends Cogn. Sci. 26, 128–141. doi: 10.1016/j.tics.2021.11.003, PMID: 34973925

[ref19] Hughes G. Desantis A. Waszak F. (2013). Mechanisms of intentional binding and sensory attenuation: the role of temporal prediction, temporal control, identity prediction, and motor prediction. Psychol. Bull. 139, 133–151. doi: 10.1037/a0028566, PMID: 22612280

[ref20] Jackson C. V. (1953). Visual factors in auditory localization. Q. J. Exp. Psychol. 5, 52–65. doi: 10.1080/17470215308416626

[ref21] Jagini K. K. (2021). Temporal binding in multisensory and motor-sensory contexts: toward a unified model. Front. Hum. Neurosci. 15:629437. doi: 10.3389/fnhum.2021.629437, PMID: 33841117 PMC8026855

[ref22] Keetels M. Vroomen J. (2008). Temporal recalibration to tactile–visual asynchronous stimuli. Neurosci. Lett. 430, 130–134. doi: 10.1016/j.neulet.2007.10.044, PMID: 18055112

[ref23] Kohlrausch A. van Eijk R. Juola J. F. Brandt I. van de Par S. (2013). Apparent causality affects perceived simultaneity. Atten. Percept. Psychophys. 75, 1366–1373. doi: 10.3758/s13414-013-0531-0, PMID: 23943500

[ref24] Lange J. Kapala K. Krause H. Baumgarten T. J. Schnitzler A. (2018). Rapid temporal recalibration to visuo-tactile stimuli. Exp. Brain Res. 236, 347–354. doi: 10.1007/s00221-017-5132-z, PMID: 29143125 PMC5809529

[ref25] Maselli A. Kilteni K. Lopez-Moliner J. Slater M. (2016). The sense of body ownership relaxes temporal constraints for multisensory integration. Sci. Rep. 6:30628. doi: 10.1038/srep30628, PMID: 27485049 PMC4971486

[ref26] McIntyre J. Zago M. Berthoz A. Lacquaniti F. (2001). Does the brain model Newton’s laws? Nat. Neurosci. 4, 693–694. doi: 10.1038/89477, PMID: 11426224

[ref27] Morein-Zamir S. Soto-Faraco S. Kingstone A. (2003). Auditory capture of vision: examining temporal ventriloquism. Cogn. Brain Res. 17, 154–163. doi: 10.1016/s0926-6410(03)00089-2, PMID: 12763201

[ref28] Pick H. L. Warren D. H. Hay J. C. (1969). Sensory conflict in judgments of spatial direction. Percept. Psychophys. 6, 203–205. doi: 10.3758/BF03207017

[ref29] Senot P. Zago M. Lacquaniti F. McIntyre J. (2005). Anticipating the effects of gravity when intercepting moving objects: differentiating up and down based on nonvisual cues. J. Neurophysiol. 94, 4471–4480. doi: 10.1152/jn.00527.2005, PMID: 16120661

[ref30] Shimojo S. (2014). Postdiction: its implications on visual awareness, hindsight, and sense of agency. Front. Psychol. 5:196. doi: 10.3389/fpsyg.2014.00196, PMID: 24744739 PMC3978293

[ref31] Spence C. Baddeley R. Zampini M. James R. Shore D. (2003). Multisensory temporal order judgments: when two locations are better than one. Percept. Psychophys. 65, 318–328. doi: 10.3758/BF03194803, PMID: 12713247

[ref33] Suzuki K. Lush P. Seth A. K. Roseboom W. (2019). Intentional binding without intentional action. Psychol. Sci. 30, 842–853. doi: 10.1177/0956797619842191, PMID: 31023161

[ref34] Umemura H. (2017). Causal context presented in subsequent event modifies the perceived timing of cause and effect. Front. Psychol. 8:314. doi: 10.3389/fpsyg.2017.00314, PMID: 28326051 PMC5339221

[ref35] Vatakis A. Ghazanfar A. A. Spence C. (2008). Facilitation of multisensory integration by the “unity effect” reveals that speech is special. J. Vis. 8, 14.1–14.1411. doi: 10.1167/8.9.14, PMID: 18831650

[ref36] Vatakis A. Spence C. (2007). Crossmodal binding: evaluating the “unity assumption” using audiovisual speech stimuli. Percept. Psychophys. 69, 744–756. doi: 10.3758/BF03193776, PMID: 17929697

[ref37] Vatakis A. Spence C. (2008). Evaluating the influence of the ‘unity assumption’ on the temporal perception of realistic audiovisual stimuli. Acta Psychol. 127, 12–23. doi: 10.1016/j.actpsy.2006.12.002, PMID: 17258164

[ref39] Vroomen J. Stekelenburg J. J. (2011). Perception of intersensory synchrony in audiovisual speech: not that special. Cognition 118, 75–83. doi: 10.1016/j.cognition.2010.10.002, PMID: 21035795

[ref40] Wallace M. T. Hairston W. D. Stein B. E. Vaughan J. W. Schirillo J. A. (2004). Unifying multisensory signals across time and space. Exp. Brain Res. 158, 252–258. doi: 10.1007/s00221-004-1899-9, PMID: 15112119

[ref9001] Warren D. H. Welch R. B. McCarthy T. J. (1981). The role of visual–auditory “compellingness” in the ventriloquism effect: Implications for transitivity among the spatial senses. Perception & Psychophysics, 30, 557–564. doi: 10.3758/BF03202010, PMID: 7335452

[ref41] Waszak F. Cardoso-Leite P. Hughes G. (2012). Action effect anticipation: neurophysiological basis and functional consequences. Neurosci. Biobehav. Rev. 36, 943–959. doi: 10.1016/j.neubiorev.2011.11.004, PMID: 22108008

[ref42] Welch R. B. Warren D. H. (1980). Immediate perceptual response to intersensory discrepancy. Psychol. Bull. 88, 638–667. doi: 10.1037/0033-2909.88.3.638, PMID: 7003641

[ref43] Zago M. McIntyre J. Senot P. Lacquaniti F. (2008). Internal models and prediction of visual gravitational motion. Vis. Res. 48, 1532–1538. doi: 10.1016/j.visres.2008.04.005, PMID: 18499213

